# Management of Pediatric Congenital Heart Disease

**DOI:** 10.3390/jcm13237340

**Published:** 2024-12-02

**Authors:** François Godart

**Affiliations:** Department of Pediatric Cardiology and Congenital Heart Disease, University of Lille, 59000 Lille, France; francois.godart@chru-lille.fr

We are pleased to present a Special Issue dedicated to pediatric cardiology. Remarkable progress has been achieved in this field over the last 20–30 years thanks to the development of new tools, the progression of interventional cardiac catheterization alongside surgery, the development of new concepts in surgery even in complex diseases such as univentricular heart disease, and the apparition of new connecting systems leading to the emergence of E-health in this discipline. This Special Issue comprises six articles covering different aspects of these topics.

Percutaneous valvuloplasty has been the treatment of choice for pulmonary valve stenosis since 1982. This disease is mainly characterized by a right ventricle (RV) pressure overload [1]. Sirico et al. (Contribution 1) study the effects of balloon dilatation on right ventricular function in a pediatric population. After dilatation, a significant decrease in the transpulmonary gradient and RV/systolic pressure ratio is noticed, relief of stenosis is effective, and mild pulmonary regurgitation is observed. However, no change is noticed in the longitudinal RV function and tricuspid annular plane systolic excursion (TAPSE) or RV free-wall longitudinal strain (RVFWLS) on speckle tracking except for the apical segments. On the other hand, the RV fractional area change improves, which is more load-dependent, expressing the combination of longitudinal and radial functions with the early recovery of circumferential fibers, while TAPSE and RVFWLS are less load-dependent [2,3]. This suggests that longitudinal function impairment persists more than 24 h after dilation and the recovery begins from the apical segments.

The new multifunctional occluder or Konar system (Lifetech) is a new device dedicated to the correction of perimembranous ventricular septal defect (pm VSD) [4,5]. However, it has multiple facets and has already been used in the closure of muscular VSD or patent arterial ducts. This device consists of a plug placed within the left-sided defect and a retention disk placed in the right-sided defect. Delivery can be achieved from both sides; the approach could be antegrade from the venous side or retrograde from the aortic side. Godart et al. (Contribution 2) present a study from in relation to the correction of pm VSD with no failure at implantation and they show that the retrograde approach is the preferred route, which is mainly associated with significantly lower irradiation and fluoroscopy time. It should also be noted than 30% of patients also had multi-fenestrated defects including Gerbode defects. Here, the results remain good with either full occlusion or a tiny intra-prosthetic shunt [6]. This device seems very promising and thus convenient for pm VSD closure.

With the development of new tools in medicine, the concept of E-health has also emerged in pediatric cardiology. E-health encompasses different applications, including telemedicine for remote consultations, follow-up, and monitoring; mobile health (the use of mobile devices, smart phones, and tablets for care); electronic health records; and artificial intelligence (AI) [7]. Padovani et al. (Contribution 3) have reviewed different aspects and applications in fetal and neonatal cardiology [8]. For example, echocardiography formation could benefit from mannequins, simulators, and tablet-based clinical cases. This could also be combined with AI to enhance the efficacy and accuracy of diagnosis. Tele-echocardiography could be useful in extensive territories (the USA and Canada) or areas with limited access to pediatric cardiology. Others applications include wearable devices allowing for the recording of ECG or pulse oximetry in children [9]. These new tools could also be useful in the prevention of neuro-developmental impairment associated with the surgical correction of congenital heart disease [10]. However, there are some limitations to E-health, as follows: the remuneration of consultations; the family access to phones and computers; data security and privacy; and some ethical considerations. E-health is thus very promising but remains a real challenge for fetal–neonatal patients with the aim of providing the highest standard of care for these patients.

With the constant development of new valves, pulmonary valve implantation in the large right ventricular outflow tract (RVOT) has also been questioned, especially after Fallot repair. Houeijeh et al. (Contribution 4) have reported use of the Sapien 3 valve using two implantation techniques—the conventional method including pre-stenting to avoid stent fracture when the Melody valve is used [11], and the modified method with implantation through a large protective sheath (Dryseal sheath, Gore) [12]. This is possible in patients with no severe stenosis and a stable landing zone of less than 29 mm. The two groups were matched, presenting similar hemodynamic conditions. The modified technique is significantly associated with a reduction in the procedure’s complexity and length, as well as using a lower dose of irradiation. No difference is noticed during follow-up concerning the valvar gradient or dysfunction, stent fracture, secondary valve replacement, infective endocarditis, or death. Indeed, the cobalt–chrome stent used in the Sapien valve has a large radial force strength and robustness compared to the iridium–platinum one used in the Melody valve, leading to the direct placement of the Sapien valve through the Dryseal sheath, which is effective if RVOT does not exceed 29 mm on balloon testing. On the other hand, pre-stenting increases the complexity of implantation, its duration, its cost, and the fluoroscopy time. For all these reasons, the modified approach through a large protective sheath is becoming more popular and is predominantly being used for pulmonary valve implantation in large native RVOT [13].

Surgical atrial septal defect (ASD) closure remains widely employed in small children below 15 kg [14,15]. Pilar et al. (Contribution 5) compare surgery and transcatheter closure in this population. The classic indications for correction are failure to thrive and right heart enlargement. In the surgical group, the defect is a bit larger and more patients have a deficient posterior rim. As expected, the length of hospital stay is shorter in the transcatheter group and the rate of procedural complications is higher in the surgical group due to the increased rate of minor complications. No major complication is reported in either group during the median follow-up of 6 months. In the same way, symptoms resolve in both groups with the exception of failure to thrive in the population of patients with associated extracardiac malformations, where the result is less effective and closure should be argued on a case-by-case basis [16]. In fact, both approaches are efficient, but surgery remains necessary for complex anatomical ASD.

The Fontan procedure initially proposed for tricuspid atresia, is now being applied for single ventricle or functional univentricular heart disease. This model consists of by-passing the right ventricle (RV) by connecting both the inferior vena cava (IVC) and superior vena cava (SVC) directly to the pulmonary arteries. Despite its efficacy [17], patients face many complications over time, such as congestive liver failure, pulmonary vascular remodeling due to non-pulsatile blood flow, kidney failure, and plastic bronchiolitis, yielding the concept of “failing Fontan” [18]. Jalal et al. (Contribution 6) performed a review of various animal models in Fontan connections, questioning surgical issues, complications, pulmonary vasculature, and the development of new therapies or assistance strategies. It has been shown that the need of a valve between the right atrium (RA) and the pulmonary artery to optimize pump effect and reduce regurgitation is not recommended. In fact, the surgical techniques have evolved a lot with time; first, a bidirectional Glenn procedure is followed by the implantation of an extracardiac conduit. The experimental work has demonstrated better results in the two-stage strategy realized with cardiopulmonary bypass [19]. Alternatives to the completion have also been proposed in sheep models using interventional catheterization techniques, such as the preparation of the connection between SVC and RA and completion by cath and implantation of a covered stent between the IVC and pulmonary arteries [20]. Animal models were also used to explore the complications related to Fontan circulation. The negative effect of initial direct atrio-pulmonary connection with energy loss (fluid) has been demonstrated in relation to increased RA volume. In another canine model, a higher expression of endothelin-1 and nitric oxide was noticed; this was explained by the loss of pulsatility on pulmonary blood flow. It has also been demonstrated that the intra-atrial lateral tunnel was associated with more atrial arrhythmias, demonstrating the need for the application of extracardiac conduits, which is largely employed at the moment. In failing Fontan, the use of a co-axial pump to drain venous blood from the vena cava to the pulmonary artery has been tested, showing an increase in cardiac output and a decrease in pulmonary vascular resistance [21]. In fact, all the experimental work has been essential to better understand the Fontan circulation and its pitfalls. Animal models remain essential to optimize the results.

In summary, the studies included in this Special Issue gather different aspects of pediatric cardiology, new tools, and new concepts, leading to the constant progression of interventional cardiac catheterization alongside the classic surgical approach. However, animal models have clearly demonstrated their necessity to improve the surgical techniques in relation to the Fontan repair. Finally, the development of telemedicine, monitoring, mobile health, electronic health records, and artificial intelligence (AI) is clearly changing the game-making concept of E-health—a real new paradigm in congenital heart disease.
